# 

**Published:** 1892-05-28

**Authors:** 


### CONTENTS.

Suffering London . . . . . . . . . . 129

Passing Topics.—Wanted—Locality . . . . . . 130

The Doctor's Armchair.—Poor Man's Gout . . . . 131

Prisons at Home and Abroad . . . . . . . 132

Medical History from the Earliest Times. By E. T. Withington, M.A., M.B.Oxon. X.—Hippocrates . . . 133

Everybody's Page . . . . . . . . . . 134

Annotations.—Trades Unionism Run Mad—An Extraordinary Lancashire Man—Another Cancer Cure . . . . . . 135

Notes and News . . . . . . . . . . 136

Around the Hospitals . . . . . . . . . 137

Scraps and Gleanings . . . . . . . . . 137

Editor's Letter Box.—Horse Civilisation in Shetland and Wales . . . . . . . . . . . . 144

The Practitioner's Mirror and Retrospect—

Medicine.—Is Laminectomy Desirable in Traumatic Compression of the Cord? . . . . . . . . . . 138

Surgery.—Colles's Fracture . . . . . . . . 139

New Drugs, Appliances, and Things Medical.—Ingrowing Toe-nails . . . . . . . . . . . 140

The Institutional Workshop—

Hospital Construction—Temporary Hospitals—Radcliffe Infirmary, Oxford . . . . . . . . . . 141

Festivals and Dinners.—The Clarence Memorial Wing at St. Mary's Hospital, 141; The Royal London Ophthalmic Hospital 142

Furniture and Fittings.—Bed Rests, 143; Bed Table . . 144

The Student of Medicine.—Appointments—Vacancies—Pass Lists.

### EXTRA SUPPLEMENT.—THE NURSING MIRROR.

En Passant.—Lectures to Nurses—The Nurses' Hostel—Random Evidence—Hospital Reports—Nottingham and Notts Nursing Association—Annual Meeting of the Workhouse Infirmary Nursing Association—Poor, and often Friendless—Blackpool Ladies' Sick Poor Association—Concert at the London Hospital lxi

Ventilation, Disinfection, and Diet. By P. Caldwell Smith, M.D. VII.—Disinfection (*continued*) . . . . lxii

Prospects of Private Nurses . . . . . . . lxiii

Appointments . . . . . . . . . . lxviii

For Reading to the Sick.—"Human Love" . . . . lxiii

Proposed Midwives Bill . . . . . . . . lxiv

Wants and Workers . . . . . . . . . lxiv

For Asylum Attendants . . . . . . . . lxv

Everybody's Opinion.—The Danger of Developing into a Tyrant . . . . . . . . . . . lxvi

Hints to Nurses . . . . . . . . . . lxvi

Where to Go . . . . . . . . . . . lxvi

Nurses' Residential Club—Notes and Queries . . lxvi

